# Decomposition Analysis of the Prevalence of Denture Use Between Rural and Urban Older Individuals With Edentulism in China: Cross-Sectional Study

**DOI:** 10.2196/48778

**Published:** 2024-12-13

**Authors:** Lei Yuan, Yuan Yuan, Haotian Ren, Feng Zhang, Zhe Zhao, Qinqin Jiang, Zhenbang Wei, Jin-hai Sun

**Affiliations:** 1 Department of Health Management Faculty of Military Health Service Naval Medical University Shanghai China; 2 Xiuwen County Bureau of Statistics GuiZhou China; 3 College of Humanities and Management Guizhou University of Traditional Chinese Medicine GuiZhou China; 4 Department of Stomatology Changhai Hospital Naval Medical University Shanghai China

**Keywords:** oral health, older adults with edentulism, edentulism, denture, decomposition analysis, China

## Abstract

**Background:**

Edentulism impacts the physical health and quality of life of older individuals. The prevalence, influencing factors, and differences in terms of edentulism in urban and rural areas of China are unclear.

**Objective:**

This study investigated the denture-wearing conditions and causes affecting older patients with edentulism in urban and rural areas of China and analyzed the differences.

**Methods:**

This cross-sectional study included the data of 5139 older individuals (age>65 years) with edentulism obtained from 23 Chinese provinces in 2018. Participants were divided into urban and rural groups based on their household registration. Factors influencing denture use in both groups were explored using binary logistic regression, while factors influencing differences in denture wearing in both groups were explored using the Fairlie decomposition model.

**Results:**

Of the 5139 participants, 67.05% (808/1205) from urban areas and 51.12% (2011/3934) from rural areas wore dentures. In the urban group, participants with a higher level of education (1-6 years: odds ratio [OR] 2.093, 95% CI 1.488-2.945; ≥7 years: OR 2.187, 95% CI 1.565-3.055) or who exercised (OR 2.840, 95% CI 2.016-3.999) preferred wearing dentures, but individuals with BMI<18.5 kg/m2 (OR 0.558, 95% CI 0.408-0.762) or widowed (OR 0.618, 95% CI 0.414-0.921) did not. In the rural group, a higher level of education (1-6 years: OR 1.742, 95% CI 1.429-2.123; ≥7 years: OR 1.498, 95% CI 1.246-1.802), living alone (OR 1.372, 95% CI 1.313-1.663), exercise (OR 1.612, 95% CI 1.340-1.940), high economic status (OR 1.234, 95% CI 1.035-1.472), residence in the eastern area (OR 2.045, 95% CI 1.723-2.427), presence of chronic diseases (1 disease: OR 1.534, 95% CI 1.313-1.793; ≥2 diseases: OR 1.500, 95% CI 1.195-1.882) were positively associated and age≥80 years (OR 0.318, 95% CI 0.247-0.408), BMI<18.5 kg/m2 (OR 0.692, 95% CI 0.590-0.813), and widowed (OR 0.566, 95% CI 0.464-0.690) or other marital status (OR 0.600, 95% CI 0.392-0.918) were negatively associated with denture use. The Fairlie decomposition model revealed that the number of chronic diseases (16.34%), education level (11.94%), region of residence (11.00%), annual income (10.55%), exercise (6.81%), and age (–0.92%) were the main factors responsible for differences between urban and rural edentulism and could explain the difference in the denture-wearing rate (58.48%) between both groups.

**Conclusions:**

Older individuals with edentulism with a higher education level and who exercise are more willing to wear dentures, while those with BMI<18.5 kg/m2 show a decreased willingness to wear dentures in both urban and rural areas in China. Controlling the number of chronic diseases, improving the education level and annual income, cultivating good exercise habits, and bridging the gap between the economic status of the east and west can narrow the differences in denture wearing among urban and rural older individuals with edentulism.

## Introduction

Oral diseases have become a public health concern worldwide [1], affecting 64.6% of the Chinese older adult population in varying degrees [2,3]. Oral diseases particularly affect older individuals and are majorly responsible for the disease burden [4,5]. Edentulism is the loss of all natural teeth, and it is 1 of the most serious oral diseases [6]. Patients with edentulism face serious oral health risks, adversely affecting their health and quality of life [7]. The absolute disease burden due to edentulism is increasing in many countries [8].

According to current research, patients with edentulism are more prone to chronic diseases (eg, hypertension [9], diabetes, chronic kidney disease, cardiovascular disease, and asthma) [10], psychological abnormalities (eg, sleep disorders) [11], and increased cognitive impairment [12]. Reportedly, edentulism increases the risk of all-cause mortality [13] and a poorer quality of life [14].

Several surveys have reported that the prevalence of edentulism among middle-aged and older Chinese individuals ranges from approximately 8% to 28% [15-19]. China is experiencing a crisis of rapid population aging. According to the Seventh National Population Census Data released by the State Council of China, in 2021, the older adult population (age>65 years) accounted for 190 million (approximately 13.5% of the total population), which is >14% in some provinces [20]. By 2050, the older adult population in China will be nearly 487 million, accounting for 34.9% of the total population. This gradual aging phenomenon indicates that the older adult population in China will suffer from serious oral health problems, such as edentulism.

Replacing missing teeth is the most effective treatment for edentulism, while dentures are the main treatment modality [21]. Particularly, older individuals with edentulism are more likely to wear dentures. Wearing dentures can effectively help restore masticatory function [22], prevent malnutrition [23], improve oral health [12], lower the incidence of cognitive impairment and mental illness [24], lower all-cause mortality [13], and improve the overall physical health of the older [12,22,25].

To the best of our knowledge, there exists a limited body of research that has reported on the use of dentures among older individuals with edentulism in China. This prevailing circumstance is regrettably unfavorable and undermines the comprehensive exploration of oral health services and the formulation of pertinent policies in the Chinese context. China, as a geographically expansive and densely populated nation, has been the subject of numerous investigations revealing an escalating pattern of health disparities between urban and rural residents in response to the nation’s burgeoning economic growth [26]. This discernible phenomenon has been substantiated in domains encompassing psychological well-being, the incidence of chronic ailments, and atmospheric pollution levels [27-29]. Curiously, the realm of oral hygiene, acknowledged as a pivotal determinant influencing the health of the older populace [30,31], has regrettably remained marginalized in the academic endeavors of Chinese scholars. Notably absent is a substantive examination of the urban-rural dichotomy in denture adoption rates among geriatric cohorts with edentulism, an omission that represents a conspicuous deficiency within China’s public health research landscape. The prevailing lacuna necessitates the exploration of causative factors underpinning the incongruities in denture usage rates among older individuals with edentulism in China. This imperative investigation can furnish the Chinese governmental apparatus with actionable policy recommendations tailored to the augmentation of oral public health endeavors. These recommendations are envisaged to play a catalytic role in ameliorating the existing health disparities between China’s urban and rural spheres, thereby aligning harmoniously with the overarching objective of mitigating health inequalities on a broader societal canvas.

To address these scholarly exigencies, this study embarked on an in-depth inquiry into the unmet requirements for denture use within the edentulous older demographic in China, as well as its main influencing factors. Furthermore, it is pertinent to acknowledge the salient socioeconomic and health differentials existing between the urban and rural populace of China [26,32-34], an observation that affects the realm of oral health. There is evidence of the complex interactions of socioeconomic inequalities, urban-rural economic differentials, and the socioenvironmental milieu across different life stages shaping inconsistent oral health outcomes [35-37].

Considering these findings, this study conducted a comparative analysis of denture use among urban and rural older individuals with edentulism in China. We analyzed the differences in denture use among urban and rural older individuals with edentulism and further identified the factors influencing those differences in order to inhibit the prevailing inconsistencies in oral health outcomes between urban and rural areas. Our aim was to provide key insights into the calibration of oral health service policies and the enhancement of the oral and holistic well-being of the older adult population in China.

## Methods

### Data Sources

This was a cross-sectional study to analyze the prevalence of denture use between rural and urban older individuals with edentulism. Data were obtained from a survey conducted by the Chinese Longitudinal Healthy Longevity Survey project team in 2018, covering 23 Chinese provinces. Multistage cluster sampling was used, and about 50% of counties, county-level cities, and municipal districts in all survey provinces were randomly selected as survey areas. These areas were divided into large and small “sample counties” for the survey. Data were collected by centralized trained investigators who were assessed to be qualified for duty.

### Ethical Considerations

The study was approved by the Research Ethics Committee of Peking University and Duke University. Written informed consent was obtained from all participants during the face-to-face interview. Data collection was approved by the Research Ethics Committee of Peking University. The data were made public after anonymous processing, and detailed design reports are available on the university’s website [38-40]. As the data analyzed are available in the public domain, separate ethical approval was not required for this study.

A total of 15,874 participants were surveyed, approved by the Research Ethics Committee of Peking University (IRB00001052-13074).

### Sample Selection

Participants needed to meet the following inclusion criteria: (1) be ≥65 years old, (2) have edentulism, and (3) answer the questionnaire completely. A total of 10,641 (67.03%) participants without edentulism were excluded from our study; 22 (0.14%) participants were excluded because they did not respond to the indicator of whether dentures should be worn; 3 (0.02%) participants aged <65 years and 69 (0.43%) participants who did not respond to basic information were also excluded. Finally, 5139 (32.37%) participants were included in this study.

#### Edentulism and Denture Use Assessments

Participants with edentulism were assessed by asking the question “How many teeth do you have (excluding dentures)?” Participants who answered 0 were considered edentulous, while those who answered anything other than 0 were excluded from this study.

Participants were asked the question “Do you wear dentures?”, with “yes” or “no” as a response.

#### Variables

Participants were divided into urban and rural groups based on China’s household registration classification (*hukou*).

With similar other studies as references, the covariates included in this study comprised 4 indicator types: demographic characteristics, lifestyle characteristics, economic status, and health status. The demographic characteristics included age, sex, education level, and marital status; the lifestyle characteristics included the residential status, any history of smoking, alcohol consumption, and exercise; the economic status included the annual income and residential region; and the health status included the BMI and chronic disease status. The variable and reference group settings for all indicators are described in detail in Table S1 in Multimedia Appendix 1.

### Statistical Analysis

The basic information was analyzed using descriptive statistical methods. Wearing dentures was used as the outcome variable, 1-way ANOVA was performed using the chi-square test, and binary logistic regression was conducted to explore the factors influencing denture use in the urban and rural groups. All factors were entered into the logistic regression analysis, and forward stepwise regression was performed.

The Fairlie decomposition model was used to perform decomposition analysis. It decomposed the difference between the urban and rural groups with edentulism and without dentures into 2 based on the difference in the observation of characteristic factors and coefficients [26,41]. Specific application examples from several studies exist [42-45]. The decomposition equation used in this study was as follows:



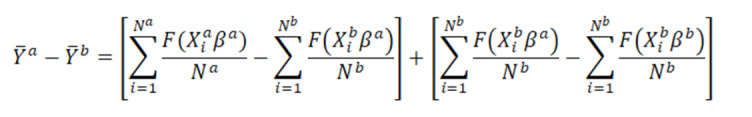



where 
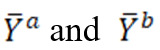
 are the average probabilities of binary outcomes for denture wearing in the urban and rural groups, respectively; F is the cumulative distribution function of the logical distribution; 
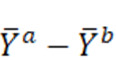
 represents the total difference due to group differences; and 
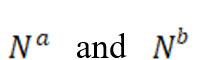
 are the sample sizes of the 2 groups of the comparator.

The first term in parentheses in the equation represents the part of the gap due to the difference in the group of observed characteristics and the part attributable to the difference in the estimated coefficient, while the second term represents the part due to the difference in the Y level [43].

## Results

### Participant Characteristics

**Figure 1:**
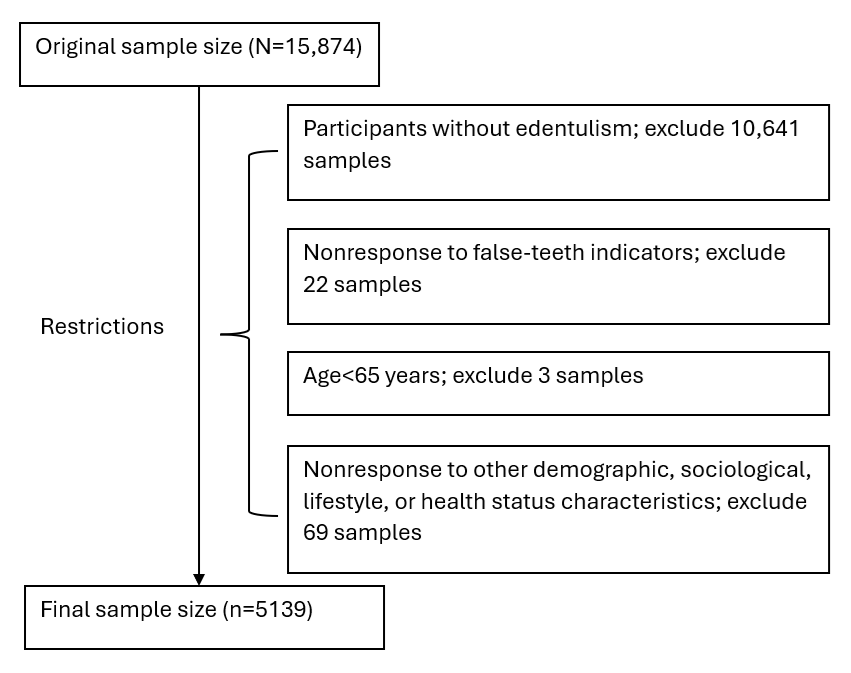
Participant selection flowchart.

The participant selection process is presented in Figure 1. Table 1 presents the results of the chi-square test for the basic participant characteristics of denture wearers and nonwearers among older individuals with edentulism in the urban and rural groups. Participants from the urban area constituted 23.45% (1205/5139) of the study population. The results revealed that except for the marital status, there were significant differences in the distribution characteristics of other factors between urban and rural participants (*P*<.05).

**Table 1:** Basic participant characteristics of denture wearers and nonwearers among older individuals with edentulism in urban and rural areas in China.

Characteristics		Urban group (n=1205), n (%)	Rural group (n=3934), n (%)
**Dentures wearers (*χ*^2^_1_=94.591; *P*<.001)**			
	No	397 (32.95)	1923 (48.88)
	Yes	808 (67.05)	2011 (51.12)
**Age (years; *χ*^2^_1_=8.353; *P*=.004)**			
	<80	140 (11.62)	589 (14.97)
	≥80	1065 (88.38)	3345 (85.03)
**Sex (*χ*^2^_1_=16.457; *P*<.001)**			
	Female	498 (41.33)	1373 (34.90)
	Male	707 (58.67)	2561 (65.10)
**BMI (kg/m^2^; *χ*^2^_3_=31.714; *P*<.001)**			
	18.5-23.9	539 (44.73)	1874 (47.64)
	<18.5	313 (25.98)	1206 (30.66)
	24.0-27.9	240 (19.92)	562 (14.29)
	≥28.0	113 (9.38)	292 (7.42)
**Education level (years; *χ*^2^_2_=141.516; *P*<.001)**			
	0	492 (40.83)	2347 (59.66)
	1-6	325 (26.97)	823 (20.92)
	≥7	388 (32.20)	764 (19.42)
**Marital status (*χ*^2^_2_=0.292; *P*=.846)**			
	Married and living with spouse	279 (23.15)	939 (23.87)
	Widowed	888 (73.69)	2876 (73.11)
	Other	38 (3.15)	119 (3.02)
**Living status (*χ*^2^_2_=203.117; *P*<.001)**			
	Living with household members	938 (77.84)	3176 (80.73)
	Living alone	136 (11.29)	681 (17.31)
	Living in an institution	131 (10.87)	77 (1.96)
**Smoking (*χ*^2^_1_=21.289; *P*<.001)**			
	No	1101 (91.37)	3397 (86.35)
	Yes	104 (8.63)	537 (13.65)
**Alcohol consumption (*χ*^2^_1_=7.936; *P*=.005)**			
	No	1098 (91.12)	3470 (88.21)
	Yes	107 (8.88)	464 (11.79)
**Exercise (*χ*^2^_1_=49.507; *P*<.001)**			
	No	861 (71.45)	3184 (80.94)
	Yes	344 (28.55)	750 (19.06)
**Annual income (*χ*^2^_3_=508.961; *P*<.001)**			
	Very low	119 (9.88)	1272 (32.33)
	Low	109 (9.05)	843 (21.43)
	Middle	162 (13.44)	504 (12.81)
	High	815 (67.63)	1315 (33.43)
**Residential region (*χ*^2^_2_=92.747; *P*<.001)**			
	Central	188 (15.60)	994 (25.27)
	Western	251 (20.83)	1049 (26.66)
	Eastern	766 (63.57)	1891 (48.07)
**Number of chronic diseases (*χ*^2^_2_=300.031; *P*<.001)**			
	0	470 (39.00)	2286 (58.11)
	1	344 (28.55)	1184 (30.10)
	≥2	391 (32.45)	464 (11.79)

### Assessment of the Proportion of Denture Wearing Across Provinces

Table S2 in Multimedia Appendix 2 shows the results of the chi-square test for the proportion of denture wearing among older individuals with edentulism by region (central, western, and eastern) and by *hukou* (urban and rural). The results revealed that 67.05% (808/1205) of participants in the urban group used dentures (95% CI 64.40-69.71), while 51.12% (2011/3934) of participants in the rural group used dentures (95% CI 49.56-52.67), and the difference was statistically significant (*χ*^2^_1_=94.591, *P*<.001). The prevalence of denture use across the various regions of China was as follows: central region, 45.26% (535/1182; 95% CI 42.42-48.10); western region, 47.92% (623/1300; 95% CI 45.20-50.64); and eastern region, 62.51% (1661/2657; 95% CI 60.67-64.36). The regional differences were statistically significant (*χ*^2^_2_=132.086, *P*<.001).

**Figure 2:**
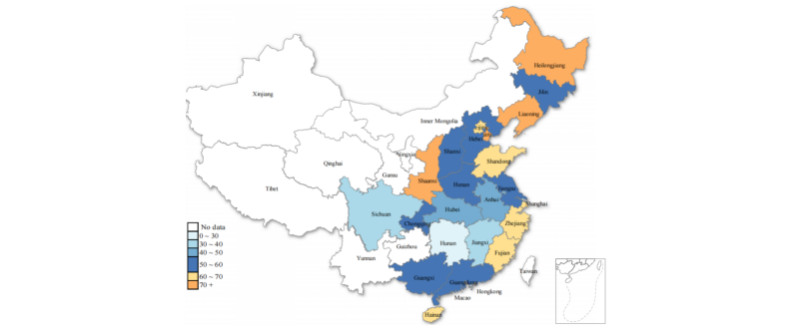
Proportion of denture use among older individuals with edentulism across 23 Chinese provinces.

Based on the survey results, we mapped the proportion of denture use among older individuals with edentulism across 23 Chinese provinces (Figure 2 and Table S3 in Multimedia Appendix 3). We observed that the highest proportion was in Liaoning (81.36%, 95% CI 74.23-88.49), while the lowest proportion was in Hunan (21.91%, 95% CI 15.57-28.05) among the provinces surveyed.

### Distribution of Denture Wearers and Nonwearers Among Older Individuals With Edentulism

Table 2 reveals the results of the chi-square test for the distribution characteristics of denture wearers and nonwearers among older participants with edentulism in the urban and rural groups. The results revealed that the distribution of certain factors among participants with dentures in urban and rural groups was significantly different; certain differences were also observed in those without dentures. Age, marital status, alcohol consumption, and exercise were significantly different among participants in urban and rural groups who were denture wearers; however, no differences were observed in denture nonwearers.

**Table 2:** Distribution of characteristics of denture wearers and nonwearers among older individuals with edentulism in urban and rural areas in China.

Characteristics		Denture wearers		Denture nonwearers	
		Urban group (n=808), n (%)	Rural group (n=2011), n (%)	Urban group (n=397), n (%)	Rural group (n=1923), n (%)
**Age (years; wearers: *χ*^2^_1_=30.186, *P*<.001; nonwearers: *χ*^2^_1_=0.009, *P*>=.924)**					
	<80	119 (14.73)	485 (24.12)	21 (5.29)	104 (5.41)
	≥80	689 (85.27)	1526 (75.88)	376 (94.71)	1819 (94.59)
**Sex (wearers: *χ*^2^_1_=2.840, *P*=.092; nonwearers: *χ*^2^_1_=7.534, *P*=.006)**					
	Female	355 (43.94)	814 (40.48)	143 (36.02)	559 (29.07)
	Male	453 (56.06)	1197 (59.52)	254 (63.98)	1364 (70.93)
**BMI (kg/m^2^; wearers: *χ*^2^_3_=6.276, *P*=.099; nonwearers: *χ*^2^_3_=17.981, *P*<.001)**					
	<18.5	162 (20.05)	449 (22.33)	151 (38.04)	757 (39.37)
	18.5-23.9	388 (48.02)	1012 (50.32)	151 (38.04)	862 (44.83)
	24.0-27.9	178 (22.03)	384 (19.09)	62 (15.62)	178 (9.26)
	≥28.0	80 (9.90)	166 (8.25)	33 (8.31)	126 (6.55)
**Education level (years; wearers: *χ*^2^_2_=82.549, *P*<.001; nonwearers: *χ*^2^_2_=25.672, *P*<.001)**					
	0	268 (33.17)	1010 (50.22)	224 (56.42)	1337 (69.53)
	1-6	244 (30.20)	550 (27.35)	81 (20.40)	273 (14.20)
	≥7	296 (36.63)	451 (22.43)	92 (23.17)	313 (16.28)
**Marital status (wearers: *χ*^2^_2_=7.918, *P*=.019; nonwearers: *χ*^2^_2_=0.334, *P*=.846)**					
	Married and living with spouse	227 (28.09)	674 (33.52)	52 (13.10)	265 (13.78)
	Widowed	557 (68.94)	1277 (63.50)	331 (83.38)	1599 (83.15)
	Other	24 (2.97)	60 (2.98)	14 (3.53)	59 (3.07)
**Living status (wearers: *χ*^2^_2_=106.039, *P*<.001; nonwearers: *χ*^2^_2_=107.303, *P*<.001)**					
	Living with household members	633 (78.34)	1600 (79.56)	305 (76.83)	1576 (81.96)
	Living alone	96 (11.88)	375 (18.65)	40 (10.08)	306 (15.91)
	Living in an institution	79 (9.78)	36 (1.79)	52 (13.10)	41 (2.13)
**Smoking (wearers: *χ*^2^_1_=21.830, *P*<.001; nonwearers: *χ*^2^_1_=5.471, *P*=.019)**					
	No	732 (90.59)	1685 (83.79)	369 (92.95)	1712 (89.03)
	Yes	76 (9.41)	326 (16.21)	28 (7.05)	211 (10.97)
**Alcohol consumption (wearers: *χ*^2^_1_=8.700, *P*=.003; nonwearers: *χ*^2^_1_=2.371, *P*=.124)**					
	No	730 (90.35)	1735 (86.28)	368 (92.70)	1735 (90.22)
	Yes	78 (9.65)	276 (13.72)	29 (7.30)	188 (9.78)
**Exercise (wearers: *χ*^2^_1_=35.094, *P*<.001; nonwearers: *χ*^2^_1_=0.087, *P*=.768)**					
	No	518 (64.11)	1512 (75.19)	343 (86.40)	1672 (86.95)
	Yes	290 (35.89)	499 (24.81)	54 (13.60)	251 (13.05)
**Annual income (wearers: *χ*^2^_3_=328.842, *P*<.001; nonwearers: *χ*^2^_3_=160.774, *P*<.001)**					
	Very low	67 (8.29)	649 (32.27)	52 (13.10)	623 (32.40)
	Low	74 (9.16)	403 (20.04)	35 (8.82)	440 (22.88)
	Middle	103 (12.75)	260 (12.93)	59 (14.86)	244 (12.69)
	High	564 (69.80)	699 (34.76)	251 (63.22)	616 (32.03)
**Residential region (wearers: *χ*^2^_2_=18.504, *P*<.001; nonwearers: *χ*^2^_2_=67.608, *P*<.001)**					
	Central	118 (14.60)	417 (20.74)	70 (17.63)	577 (30.01)
	Western	168 (20.79)	455 (22.63)	83 (20.91)	594 (30.89)
	Eastern	522 (64.60)	1139 (56.64)	244 (61.46)	752 (39.11)
**Number of chronic diseases (wearers: *χ*^2^_2_=144.694, *P*<.001; nonwearers: *χ*^2^_2_=128.438, *P*<.001)**					
	0	298 (36.88)	1012 (50.32)	172 (43.32)	1274 (66.25)
	1	231 (28.59)	708 (35.21)	113 (28.46)	476 (24.75)
	≥2	279 (34.53)	291 (14.47)	112 (28.21)	173 (9.00)

### Logistic Regression Results

The results of binary logistic regression for the multivariate analysis of older individuals with edentulism in the urban and rural groups who were denture wearers are presented in Table 3. The results revealed that in the urban group, a higher level of education (1-6 years: odds ratio [OR] 2.093, 95% CI 1.488-2.945; ≥7 years: OR 2.187, 95% CI 1.565-3.055) and who exercised (OR 2.840, 95% CI 2.016-3.999) were protective factors for denture use in older individuals with edentulism, while BMI<18.5 kg/m^2^ (OR 0.558, 95% CI 0.408-0.762) and a widowed marital status (OR 0.618, 95% CI 0.414-0.921) were risk factors for denture use in older individuals with edentulism. In the rural group, a higher level of education (1-6 years: OR 1.742, 95% CI 1.429-2.123; ≥7 years: OR 1.498, 95% CI 1.246-1.802), living alone (OR 1.372, 95% CI 1.313-1.663), exercise (OR 1.612, 95% CI 1.340-1.940), a high economic status (OR 1.234, 95% CI 1.035-1.472), residence in the eastern area (OR 2.045, 95% CI 1.723-2.427), the presence of chronic diseases (1 disease: OR 1.534, 95% CI 1.313-1.793; ≥2 diseases: OR 1.500, 95% CI 1.195-1.882) were protective factors for denture use in older individuals with edentulism, while age ≥80 years (OR 0.318, 95% CI 0.247-0.408), BMI <18.5 kg/m^2^ (OR 0.692, 95% CI 0.590-0.813), and widowed (OR 0.566, 95% CI 0.464-0.690) or other marital status (OR 0.600, 95% CI 0.392-0.918) were risk factors for dentures in older individuals with edentulism.

**Table 3:** Logistic regression results of denture use in older individuals with edentulism in urban and rural areas in China.

	Variables	Urban group		Rural group	
		*P* value	OR^a^ (95% CI)	*P* value	OR (95% CI)
**Age (years)**					
	<80	—^b^	1.000	—	1.000
	≥80	.439	0.804 (0.462-1.398)	<.001	0.318 (0.247-0.408)
**Sex**					
	Female	—	—	—	—
	Male	.072	1.327 (0.975-1.806)	.978	0.998 (0.839-1.186)
**BMI (kg/m^2^)**					
	18.5-23.9	—	1.000	—	1.000
	<18.5	<.001	0.558 (0.408-0.762)	<.001	0.692 (0.590-0.813)
	24.0-27.9	.954	0.989 (0.685-1.429)	.003	1.384 (1.113-1.721)
	≥28.0	.781	0.935 (0.582-1.502)	.847	1.027 (0.782-1.350)
**Education level (years)**					
	0	—	1.000	—	1.000
	1-6	<.001	2.093 (1.488-2.945)	<.001	1.742 (1.429-2.123)
	≥7	<.001	2.187 (1.565-3.055)	<.001	1.498 (1.246-1.802)
**Marital status**					
	Married and living with spouse	—	1.000	—	1.000
	Widowed	.018	0.618 (0.414-0.921)	<.001	0.566 (0.464-0.690)
	Other	.158	0.562 (0.253-1.251)	.019	0.600 (0.392-0.918)
**Living status**					
	Living with household members	—	1.000	—	1.000
	Living alone	.565	1.138 (0.733-1.765)	.001	1.372 (1.131-1.663)
	Living in an institution	.375	0.826 (0.542-1.259)	.849	1.048 (0.645-1.705)
**Smoking**					
	No	—	1.000	—	1.000
	Yes	.389	1.251 (0.751-2.083)	.574	1.066 (0.853-1.331)
**Alcohol consumption**					
	No	—	—	—	—
	Yes	.966	1.011 (0.618-1.653)	.282	1.134 (0.902-1.425)
**Exercise**					
	No	—	1.000	—	1.000
	Yes	<.001	2.840 (2.016-3.999)	<.001	1.612 (1.340-1.940)
**Annual income**					
	Very low	—	1.000	—	1.000
	Low	.123	1.590 (0.882-2.868)	.608	1.053 (0.864-1.284)
	Middle	.387	1.262 (0.745-2.140)	.051	1.263 (0.999-1.598)
	High	.055	1.520 (0.991-2.332)	.019	1.234 (1.035-1.472)
**Residential region**					
	Central	—	1.000	—	1.000
	Western	.795	1.059 (0.689-1.626)	.648	1.046 (0.862-1.269)
	Eastern	.230	1.252 (0.868-1.808)	<.001	2.045 (1.723-2.427)
**Number of chronic diseases**					
	0	—	1.000	—	1.000
	1	.918	1.017 (0.740-1.398)	<.001	1.534 (1.313-1.793)
	≥2	.164	1.253 (0.912-1.722)	<.001	1.500 (1.195-1.882)

^a^OR: odds ratio.

^b^Not applicable.

### Decomposition Analysis Results

Tables 4 and 5 reveal the differential decomposition of denture-wearing prevalence among older individuals with edentulism in the urban and rural groups. The results revealed that the explanatory degree of the model was 58.48%, indicating that this study could explain 58.48% of the difference in the denture-wearing rate between urban and rural older individuals with edentulism, and the remaining 41.52% accounted for urban and rural characteristics and unincluded observation indicators. The explainable part reveals that the main factors contributing to this difference were the number of chronic diseases (16.34%), education level (11.94%), residence region (11.00%), annual income (10.55%), exercise (6.81%), and age (–0.92%).

**Table 4:** Terms of decomposition in the Fairlie decomposition model.

Terms of decomposition	Denture use
Difference	0.15935487
Explained (%)	0.09317312 (58.48%)
Nonexplained (%)	0.06616842 (41.52%)

**Table 5:** Results of the explainable part of the Fairlie decomposition model (contribution % shown).

Covariates		*P* value	β	Contribution (95% CI)
**Demographic information**				
	Age	<.001	–0.0014637	–0.92 (–0.0022267 to –0.0007007)
	Sex	.299	0.0009324	0.59 (–0.0008272 to 0.0026921)
	Education level	<.001	0.0190212	11.94 (0.0140899 to 0.0239526)
	Married status	.168	0.0003782	0.24 (–0.0001591 to 0.0009156)
**Lifestyle status**				
	Living status	.018	0.0028159	1.77 (0.0004876 to 0.0051442)
	Smoking	.468	–0.0005955	–0.37 (–0.0022039 to 0.0010129)
	Alcohol consumption	.412	–0.0004585	–0.29 (–0.0015550 to 0.0006380)
	Exercise	<.001	0.0108532	6.81 (0.0084449 to 0.0132615)
**Economic status**				
	Annual income	<.001	0.0168183	10.55 (0.0081524 to 0.0254843)
	Residence region	<.001	0.0175302	11.00 (0.0138448 to 0.0212155)
**Health status**				
	BMI	.408	0.0006835	0.43 (–0.0009352 to 0.0023021)
	Number of chronic diseases	<.001	0.0260315	16.34 (0.0194942 to 0.0325688)

## Discussion

### Principal Findings

Our study revealed that 54.86% of Chinese older individuals with edentulism wear dentures, among which the denture-wearing rate of individuals with edentulism in urban areas is higher than that in rural areas, indicating that older individuals with edentulism in urban areas focus on their oral problems. In terms of regional distribution, the denture-wearing rate in the eastern region was the highest, followed by the western and central regions. Studies have reported that this is probably due to the higher health literacy level among older individuals in the eastern region and their active awareness and behavior in promoting oral health. Additionally, the higher economic level of residents in the eastern region provide a certain economic basis for the residents to obtain good oral health care. Thus, more adequate educational and medical resources help provide better access to denture-related treatment services for older individuals [46-49].

Our study revealed that age, sex, the BMI, and the education level are significant factors affecting the urban-rural distribution of older patients with edentulism (*P*<.05). Sex, the BMI, and the education level mainly affect the urban-rural distribution of older patients with edentulism, although the urban-rural distribution factors influencing denture-wearing older patients with edentulism are mainly age, the education level, and the marital status. Further analysis revealed that the education level is a protective factor for denture wearing among older patients with edentulism in urban and rural areas and that a higher education level could improve health literacy, the awareness of health maintenance, and the promotion of healthy behavior, leading to better use of medical resources, such as oral health clinics and oral health care aids, to promote their oral health [48,50]. Previous studies [51-53] have reported that the number of teeth can affect an individual’s choice of food and nutrient intake, and individuals without teeth are more likely to have a lean physique. In this study, a BMI of <18.5 kg/m^2^ was a relevant factor for denture wearing in older individuals with edentulism (urban areas: OR 0.558, 95% CI 0.408-0.762, *P*<.001; rural areas: OR 0.692, 95% CI 0.590-0.813, *P*<.001), which might be because denture use can improve the oral health of patients with edentulism, prompting them to choose more food varieties and have a more comprehensive nutritional intake, while denture nonwearers will be malnourished due to poor oral health, limited food options, and aggravated leanness.

Lifestyle habits, such as smoking and alcohol consumption, influence the use of dentures among patients with edentulism [54-56] and significantly impact the urban-rural distribution of older patients with edentulism and older patients wearing dentures (*P*<.05); the effects of the living status and exercise on the urban-rural distribution of older patients with edentulism and older patients wearing dentures are also significant (*P*<.05). However, the effects of alcohol consumption and exercise on the urban-rural distribution differences among older individuals with edentulism who do not wear dentures are not significant. Although previous studies have reported that smoking and alcohol consumption affect an individual’s oral health literacy and healthy behavior, this study did not demonstrate significant healthy behaviors among urban and rural patients with edentulism who wore dentures. Furthermore, exercise was a protective factor for denture use in older patients with edentulism (urban areas: OR 2.840, 95% CI 2.016-3.999, *P*<.001; rural areas: OR 1.61295% CI 1.340-1.940, *P*<.001). Older individuals who exercise regularly are more aware of health maintenance, have higher health requirements, and are more willing to wear dentures to maintain their quality of life and health status.

According to the provisions of the Basic Medical Insurance System for Chinese Residents, the cost of a denture for patients with edentulism is not fully reimbursed, with most of the expense being borne by the patient. Similar to previous studies [57-59], we found that the socioeconomic status and household economic status have a significant impact on dental service use. Our results revealed that the economic status and area of residence are strongly associated with the inequality of the urban-rural distribution of older patients with edentulism in China and that their impact on the difference in the urban-rural distribution of older patients with edentulism in China is significant regardless of whether the patients are denture wearers. For older patients with edentulism from rural areas, a high annual family income and residence in the eastern region were protective factors for wearing a denture. Older individuals living in urban areas have a higher and more stable financial income after retirement compared to older individuals from rural areas. Additionally, urban residents have a higher percentage of commercial health insurance than rural residents, thereby providing favorable financial support to urban patients with edentulism to obtain dentures [60,61]. In recent years, the centralized procurement policy of medical consumables has been implemented in China, and regarding oral equipment, this can significantly lower the medical burden of providing dentures to patients with edentulism and increase the proportion of dentures provided to older patients with edentulism from rural areas, thus reducing the urban-rural inequality in denture use.

Islas-Granillo et al [62] reported that chronic diseases severely impact the oral health of older adults and that older adults with multiple moderate chronic diseases are more likely to experience edentulism [54,62,63]. This study reported that regardless of whether dentures are worn, the number of chronic diseases significantly impacts the urban-rural distribution differences in older patients with edentulism. The number of chronic diseases had no significant impact on denture wearing in older patients with edentulism from urban areas but was a protective factor for older patients with edentulism from rural areas who wore dentures. The reason probably is that older patients with edentulism with chronic diseases frequently visit medical institutions, their health literacy and willingness for health maintenance are stronger, and they are in a position to maintain their oral health more actively. Wu et al [63] reported that because of behaviors, such as frequent physical examinations or hospital visits, older adults with chronic diseases have better access to health resources, including dental examinations and treatment, than those without chronic diseases.

We performed a decomposition analysis using the Fairlie model to investigate factors influencing the urban-rural differences in denture wearing among older patients with edentulism in China, which revealed that age, education level, exercise, annual household income, area of residence, and number of chronic diseases are associated with urban-rural differences in denture wearing.

### Recommendations

Based on the differences in the denture-wearing rate between older patients with edentulism from urban and rural areas, this study proposed targeted policy recommendations to improve the denture-wearing rate and to promote equity in urban and rural oral health. First, we need to focus on patients with aphthous ulcers who have poor oral health literacy and a low education level. It is recommended that Chinese family physicians give full play to health education and guidance to create oral health awareness and motivate such patients to develop good oral health habits. Second, due to the differences in family income and regional economic development, it is necessary to focus on older patients with edentulism from rural or underdeveloped areas. The government should increase the investment in health care to lower the cost of dentures for patients with edentulism, thereby facilitating patients to easily avail of denture treatment. Finally, we recommend actively improving oral health education, maintaining oral hygiene, and encouraging residents to develop an exercise routine to maintain oral health. In summary, the oral health situation of older individuals with edentulism in China is serious, and the denture-wearing rate needs to be improved. The government should adopt measures to promote oral health, increase oral health medical funding, expand basic social medical coverage, and implement a centralized procurement policy for oral medical materials to reduce the medical burden of older patients with edentulism who require dentures, thereby improving their denture-wearing rate and promoting their oral health.

### Limitations

This study has several limitations. First, older patients with edentulism are widely distributed across China; however, this study only investigated a small number of individuals in certain provinces of the country and did not cover the entire older adult population. Second, denture use among older patients with edentulism is influenced by several factors, only some of which were analyzed in this study. Third, even though the self-reported number of teeth and denture use in this study were credible, in the follow-up studies, clinical diagnosis could be made with professional instruments. Fourth, as a cross-sectional study, the causal relationship could not be elucidated, and it is suggested that a follow-up study be conducted prospectively.

### Conclusion

Herein, we analyzed the rate of denture wear and explored the existence and causes of the differences in the denture-wearing rate among older individuals with edentulism in urban and rural China. Studies have demonstrated that the proportion of denture wearers among older patients with edentulism in rural areas is significantly lower than that in urban areas. The government needs to formulate corresponding measures in terms of the number of chronic diseases, annual household income, region of residence, education level, and exercise to bridge the gap in the denture-wearing rates among older patients with edentulism in rural and urban China. The findings of this study can provide a theoretical basis for developing relevant health policies to minimize the difference in denture-wearing rates between older patients with edentulism in urban and rural areas.

## Appendix

Multimedia Appendix 1 Definition and measurement of variables.

Multimedia Appendix 2 Prevalence of denture wearing among older individuals with edentulism by region (central, western, and eastern) and by hukou (urban and rural).

Multimedia Appendix 3 Proportion of denture use among older individuals with edentulism across 23 Chinese provinces.

## Abbreviations

OR: Odds ratio

## Data Availability

The data sets analyzed in this study can be found on the Peking University Open Research Data website [40].
