# Supplementary material for: Decomposition Analysis of the Prevalence of Denture Use Between Rural and Urban Older Individuals With Edentulism in China: Cross-Sectional Study
Source: Interact J Med Res. 2024 Dec 13;13:e48778. doi: 10.2196/48778 (PMC11681290; doi:10.2196/48778)
Supplement: Multimedia Appendix 1 [file ijmr_v13i1e48778_app1.doc]

| Type | Name | Assignment |
| --- | --- | --- |
| Dependent variable | Wear denture | No=0, Yes=1 |
| Grouping variable | Hukou | Urban=0, Rural=1 |
| Sociological demographic characteristics | Age | <80=0, ≥80=1 |
|  | Gender | Female=0, Male=1 |
|  | Education level (school of years) | 0=0, (1-6) =1, ≥7=2 |
|  | Marital status | 0=Married and living with spouse, 1=Widowed, 2=Other* |
| lifestyle characteristics | Living status | Living with household members=0, Living alone=1, Living in an institution=2 |
|  | Smoking | Yes=1, No=0 |
|  | Drinking | Yes=1, No=0 |
|  | Exercise | Yes=1, No=0 |
| Economic status | Annual income (RMB) | Very low=0 (income<10,000), low=1 (10,000≤income<30,000), Moderate=2 (30,000≤income <50,000), High=3 (income≥50,000) |
|  | Residence region+ | Central =0, Western =1, Eastern =2 |
| Health status and security | BMI (kg/m^2^) | 18.5-23.9=0, <18.5=1, 24.0-27.9=2, ≥28.0=3 |
|  | Number of chronic diseases# | 0=0, 1=1, ≥2=2 |

0: Reference group.

*: Other married status includes married but not living with spouse, divorce, or never married.

+: Residence region: this study includes 23 provinces of China mainland. The central region includes 8 provinces: Shanxi, Jilin, Heilongjiang, Anhui, Jiangxi, Henan, Hubei and Hunan; The western region includes 4 provinces: Guangxi, Chongqing, Sichuan and Shaanxi; The eastern region includes 11 provinces: Beijing, Tianjin, Hebei, Liaoning, Shanghai, Jiangsu, Zhejiang, Fujian, Shandong, Guangdong and Hainan.

#: Chronic diseases include hypertion, heart disease, diabetes, stroke and cerebrovascular disease, cancer, prostatic diseases(male) and parkinson.
