# Supplementary material for: Decomposition Analysis of the Prevalence of Denture Use Between Rural and Urban Older Individuals With Edentulism in China: Cross-Sectional Study
Source: Interact J Med Res. 2024 Dec 13;13:e48778. doi: 10.2196/48778 (PMC11681290; doi:10.2196/48778)
Supplement: Multimedia Appendix 2 [file ijmr_v13i1e48778_app2.doc]

| Variable | | Total [n (%)] | No | Yes | Prevalence of wear denture (95%CI) | 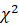 | *P* |
| --- | --- | --- | --- | --- | --- | --- | --- |
| Regional | Central | 1182 (23.00) | 647 | 535 | 45.26 (42.42,48.10) | 132.086 | <0.001 |
|  | Western | 1300 (25.30) | 677 | 623 | 47.92 (45.20,50.64) |  |  |
|  | Eastern | 2657 (51.70) | 996 | 1661 | 62.51 (60.67,64.36) |  |  |
| Hukou | Urban | 1205 (23.45) | 397 | 808 | 67.05 (64.40,69.71) | 94.591 | <0.001 |
|  | Rural | 3934 (76.55) | 1923 | 2011 | 51.12 (49.56,52.67) |  |  |
| Total | | 5139 (100.0) | 2320 | 2819 | 54.86 (53.49,56.22) |  |  |
