# Supplementary material for: Decomposition Analysis of the Prevalence of Denture Use Between Rural and Urban Older Individuals With Edentulism in China: Cross-Sectional Study
Source: Interact J Med Res. 2024 Dec 13;13:e48778. doi: 10.2196/48778 (PMC11681290; doi:10.2196/48778)
Supplement: Multimedia Appendix 3 [file ijmr_v13i1e48778_app3.doc]

| Provinces | Sample sizes | Wear denture sizes | Proportion of wear denture [95%CI (%)] |
| --- | --- | --- | --- |
| Beijing | 167 | 112 | 67.07 (59.86,74.27) |
| Tianjin | 31 | 22 | 70.97 (54.04,87.89) |
| Hebei | 40 | 22 | 55.00 (38.39,71.11) |
| Shanxi | 37 | 21 | 56.76 (40.01,73.50) |
| Inner Mongolia | 0 | 0 | No data |
| Liaoning | 118 | 96 | 81.36 (74.23,88.49) |
| Jilin | 53 | 30 | 56.60 (42.81,70.40) |
| Heilongjiang | 75 | 54 | 72.00 (61.60,82.40) |
| Shanghai | 163 | 109 | 66.87 (59.57,74.17) |
| Jiangsu | 701 | 415 | 59.20 (55.55,62.85) |
| Zhejiang | 212 | 131 | 61.79 (55.20,68.39) |
| Anhui | 168 | 69 | 41.07 (33.56,48.59) |
| Fujian | 107 | 70 | 65.42 (56.26,75.58) |
| Jiangxi | 93 | 29 | 31.18 (21.59,40.77) |
| Shandong | 669 | 409 | 61.14 (57.43,64.84) |
| Henan | 463 | 237 | 51.19 (46.62,55.76) |
| Hubei | 115 | 56 | 48.70 (39.42,57.97) |
| Hunan | 178 | 39 | 21.91 (15.57,28.05) |
| Guangdong | 299 | 179 | 59.87 (54.28,65.45) |
| Guangxi | 614 | 311 | 50.65 (46.69,54.62) |
| Hainan | 150 | 96 | 64.00 (56.23,71.77) |
| Chongqing | 186 | 100 | 53.76 (46.53,61.00) |
| Sichuan | 451 | 176 | 39.02 (34.51,43.54) |
| Guizhou | 0 | 0 | No data |
| Yunnan | 0 | 0 | No data |
| Tibet | 0 | 0 | No data |
| Shaanxi | 49 | 36 | 73.47 (60.66,86.28) |
| Gansu | 0 | 0 | No data |
| Qinghai | 0 | 0 | No data |
| Ningxia | 0 | 0 | No data |
| Xinjiang | 0 | 0 | No data |
| Taiwan | 0 | 0 | No data |
| Hongkong | 0 | 0 | No data |
| Macao | 0 | 0 | No data |
